# Isolation and characterization of porcine parvovirus in Vietnam

**DOI:** 10.14202/vetworld.2024.1530-1537

**Published:** 2024-07-13

**Authors:** T. T. Hang Trinh, V. Tan Do, V. Khien Do, Hung Vu-Khac

**Affiliations:** 1 Department of Biotechnology, Institute of Veterinary Research and Development of Central Vietnam, Nha Trang City, Vietnam; 2 Department of Virology, Institute of Veterinary Research and Development of Central Vietnam, Nha Trang City, Vietnam

**Keywords:** isolation, phylogenetic tree, porcine parvovirus, sow, virulence

## Abstract

**Background and Aim:**

No study has successfully isolated parvovirus in Vietnam. This study aimed to isolate and characterize parvovirus strains indigenous in Vietnam for vaccine development against porcine parvovirus (PPV).

**Materials and Methods:**

We collected serum and stillbirth samples from six provinces in Vietnam, and PPV-positive samples were identified using a polymerase chain reaction. Parvovirus isolation was attempted using the PK-15 cells maintained in a minimum essential medium supplemented with 5% fetal bovine serum and 1% antibiotics (Penicillin-streptomycin). The cells were incubated at 37°C with 5% CO_2_. Virulence experiments were conducted on white primiparous sows to evaluate the virulence of the PPV strain through hemagglutination inhibition (HI) titers and fetus lesions.

**Results:**

We analyzed 360 serum and 32 stillbirth (liver and lungs) samples, revealing that 32/392 (8.2% ) of them were PPV-positive, all belonging to PPV1. Thirty-two PPV-positive samples were successfully isolated, with 100% identity as VP2 sequences. The phylogenetic tree revealed a close relationship with the Kresse strain (isolated from Canada in 1996) and the PPV1-0225-L-SD strain (isolated from China in 2022). Two PPV isolates (VC5 from Dongnai and TX7 from Thanhhoa) that exhibited high 50% tissue culture infectious dose titers were selected for the virulence experiment. On day 21, after injection, the HI antibody titers ranged from 10log_2_ to 12log_2_. On day 90, 71%–80% of fetuses were mummified.

**Conclusion:**

This study showed that the PPV infection rate in Vietnam was 8.2%. Thirty-two isolates belonged to PPV1. Two PPV strains, VC5 and TX7, were determined to be highly virulent by the results of HI titers after injection into gilts. VC5 and TX7 were determined to be good candidates for further research on PPV vaccines.

## Introduction

Porcine parvovirus (PPV) was first identified in 1967 by Cartwright and Huck [1]. The virus infects all types of pig herds globally. PPV, the leading cause of pig embryo and fetus death, is responsible for complications including stillbirths, delayed estrus, and mummification [2]. At least seven distinct genetic variants (PPV1–PPV7) of porcine parvoviruses exist [3]. A current taxonomy recommended by the International Committee on Taxonomy of Viruses classifies PPV1 as *Protoparvovirus*; PPV2 and PPV3 as *Tetraparvovirus*; PPV4, PPV5, and PPV6 as *Copiparvovirus*; and PPV7 as Chapparvovirus [3, 4]. The PPV genome is a 5.2 kb single-stranded DNA molecule with terminal palindromic sequences. The genome contains two open reading frames (ORFs): One ORF encodes the structural protein (or VP), whereas the other encodes the nonstructural protein (or NSP). PPV generates an icosahedral, unenveloped capsid by combining multiple copies of VP1, VP2, and VP3. Proteins VP1 and VP2 are translated from different start codons within the same reading frame, and VP3 is derived from VP2 through proteolytic cleavage [5, 6]. The VP2 capsid protein serves as a significant antigenic site for PPV, eliciting neutralizing antibodies against the virus. Knowledge of VP2 is crucial for diagnosing PPV and administering immunological prevention [7, 8]. Based on VP2 gene analysis, distinct subgroups of PPV were identified [9].

PPV has been known since 1967 [1]. However, recent studies have shown that PPV has a relatively high evolutionary rate, ranging from 10^−5^ to 10^−4^ substitution sites^−1^. year^−1^ [10]. Consequently, vaccine protection may decrease over time. In a recent study, three commercial Parvo vaccines (based on strain NADL-2) had 64%–92% of live fetuses and 8%–36% mummified fetuses after being challenged with a PPV-27a strain [11]. Reinforcing pathogen monitoring for PPV is essential. On the other hand, Vietnam is a nation in a tropical temperature zone, where rates of speciation are higher than in other climatic zones [12]. Studies on Vietnamese parvovirus’s characteristics and gene analysis are essential.

Most studies employed an antibody or polymerase chain reaction (PCR) test for PPV identification and infection rate determination [13]. Most studies focused on using an antibody test or PCR reaction to identify PPV and determine the infection rate [13]. Data on PPV strains’ virulence and growing conditions in Vietnam are scarce. Vietnam has imported the PPV vaccine to prevent PPV infection. No study has been conducted to determine if these vaccines are suitable for virus strains circulating in Vietnam.

Our study isolated PPV strains from sows in various geographical locations within Vietnam. VP2 sequences were compared to other PPV reference strains to distinguish the current isolates. Gilts were used to assess the virulence of vaccine candidates. Further vaccine development research in Vietnam would greatly benefit from the isolation and characterization of PPV strains.

## Materials and Methods

### Ethical approval

This study was approved by the Animal Ethics Committee of Nong Lam University (NLU), Ho Chi Minh City, Vietnam (Approval number: NLU-230313).

### Study period and location

**Figure-1:**
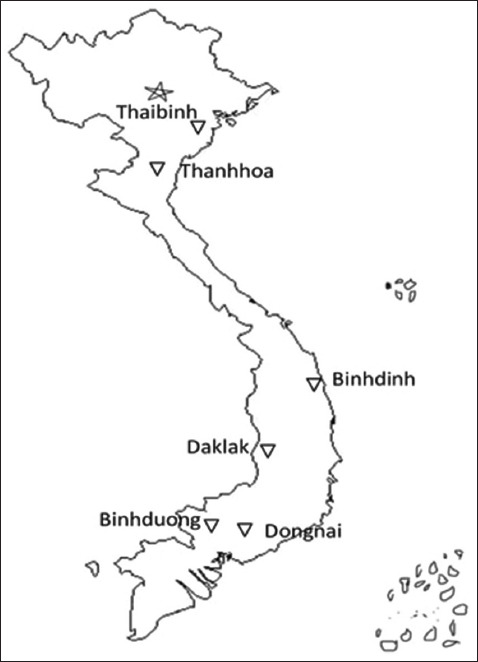
Sample collection map [Source: https://thcshoanghiep.edu.vn].

The study was conducted from January to December 2022. Serum and stillbirth samples were obtained from pig farms in six Vietnamese provinces: Thaibinh and Thanhhoa (Northern Vietnam), Binhdinh and Daklak (Central Vietnam), and Dongnai and Binhduong (Southern Vietnam) (Figure-1).

### Sample collection

We collected 360 serum and 32 stillbirth (liver and lungs) samples from farms with a history of parvovirus infection or reproductive failure. The samples were transported on ice to the Department of Biotechnology, Institute of Veterinary Research and Development of Central Vietnam (IVRD) for testing.

### PPV detection and typing identification

Total DNA was extracted using the MagMAX Viral Pathogen Kit (Thermo Fisher Scientific, USA). The detection of parvoviruses is based on the amplification of a 330 bp gene fragment (1453–1782) from the NS1 gene, which is highly homologous among PPV strains. The PCR experiment was conducted as previously described [14]. PPV1–PPV7 were classified using the PCR technique, which was evaluated as described by Kim *et al*. [15]. Positive samples were confirmed by sequencing.

### Cell culture and parvovirus isolation

The samples were tested for porcine reproductive and respiratory syndrome (PRRS), classical swine fever (CSF), and porcine circovirus (PCV) presence. This study was conducted at the Department of Diagnosis of Animal and Aquatic Diseases, IVRD. PPV was isolated using negative samples devoid of PRRS, CSF, and PCV.

Livers and lungs from stillbirths were homogenized in a 1:10 ratio using phosphate-buffered saline (PBS) solution. Centrifugation of 10% organ suspensions for 10 min at 4°C and 700× *g* was performed. The serums and 10% organ supernatants from stillbirths were filtrated through a sterile filter of 0.2 μm. PK-15 cell monolayers were treated with 1 mL of the filtrate solution. The virus was isolated with PK-15 cells maintained in minimum essential medium (Gibco, USA) supplemented with 5% fetal bovine serum and 1% antibiotics (Penicillin-Streptomycin). The PK-15 cells after viral infection were incubated at 37°C and 5% CO_2_. Cytopathic effects (CPE) were observed daily for 5 days. The virus on day 5 was harvested using three freeze-thaw cycles. Before use, cellular debris was removed by centrifugation at 2800× *g* for 20 min at 4°C. Ten percent of the first generation’s culture was carried over into the second. Furthermore, 10% of the second generation’s culture was passed down to the third. The growth of the virus was quantified using real-time PCR in accordance with the methodology outlined by Miao *et al*. [16].

### Standard curve for the quantification of viral copy number

The standard curve was constructed as described by Miao *et al*. [16] with some modifications (vector and DNA extraction kit). NS1 fragment (142 bp) was amplified with primers NS1-F: 5’-AGCCAAAAATGCAAACCCCAATA-3,’ NS1-R: 5’-CTCCACGGCTCCAAGGCTAAAG-3.’ The PCR product was inserted into pGE^M^®-T Vector (Promega, USA). After the culture was increased in *E. coli* DH5α host bacteria, the recombinant plasmid was purified using the QIAprep Spin Miniprep Kit, Germany. The concentration of the recombinant plasmid was measured using an ultraviolet/Vis photometer (BioPhotometer plus - Eppendorf, Germany). Using the DNA Copy Number Calculator on Thermo Scientific Web Tools, the DNA copy number was calculated based on the concentration of the recombinant plasmid. The recombinant plasmid was used as a template and was 10-fold serially diluted with sterile water. The cycle threshold values from real-time PCR results at each dilution concentration were noted to construct a standard curve.

### VP2 phylogenetic tree

The VP2 fragment was obtained using a PCR assay with the primers VP2F: 5’-CGAGGATCCATGAGTGAAAAT-3’ containing a *BamHI* site and VP2R: 5’-GCTGTCGACCTAGTATAATTTTCTT-3’ containing a *SalI* site. The PCR method for amplification of the VP2 gene was performed as described by Xu *et al*. [17]. PCR products were subcloned into the plasmid pGEMT (Promega). The vector containing the VP2 gene was sequenced using primers T7 and SP6. VP2 sequences were aligned using ApE software. The phylogenetic tree was constructed using the MEGA program version 6.0 with the neighbor-joining method [18] and 1000 bootstrap replicates [19]. The results of VP2 sequence analysis determined the phylogenetic relationships between Vietnamese isolates and other PPV reference strains.

### Virulence in gilts

The 50% tissue culture infectious dose (TCID_50_) titers of PPV isolates were determined following the procedure of Ramakrishnan [20]. Based on this outcome, two PPV isolates with high TCID_50_ titers were chosen for the virulence experiment.

Nine white primiparous sows, 11 months of age, were randomly assigned to three groups. Three gilts for each group and were maintained apart during the experiment. Before infection, they were all negative for PPV antibodies based on the results of the hemagglutination inhibition (HI) test [21]. They tested negative for PRRS, CSF, and PCV viruses.

On day 40 of gestation, gilts were inoculated with their designated viruses. Groups 1 and 2 were inoculated with isolates having high TCID_50_ titers. Group 3 received a PBS inoculation as their control. 10^6^ TCID_50_/mL was used to prepare the virus. Each gilt was given 4.0 mL of the virus, half intranasally and half intramuscularly. Blood samples were obtained on the specified days: days 0, 7, 14, 21, and 49 post-infection. The HI test was used to analyze the collected serums. On day 90, gilts were euthanized for fetal delivery, and a cesarean section was performed to record fetal lesions. PPV presence in fetal lungs and livers was investigated using PCR.

## Results

### PPV detection and typing identification

A total of 360 serum samples from sows with symptoms of reproductive dysfunction or those on the farm with a history of parvovirus infection and 32 stillbirths with signs of dryness and dehydration were collected in Vietnam.

**Figure-2:**
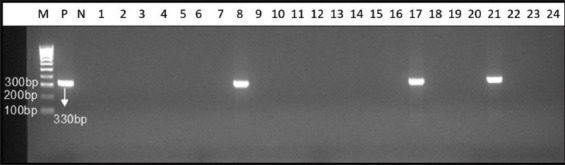
Detection of porcine parvovirus by polymerase chain reaction. M=100bp deoxyribonucleic acid ladder, P=Positive control, N=Negative control, Lane 1–24=Samples.

**Figure-3:**
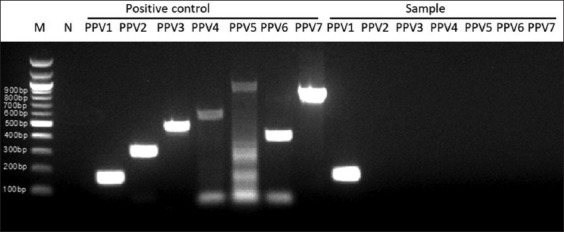
Polymerase chain reaction for PPV type detection. M=100bp deoxyribonucleic acid ladder, N=Negative control, Positive control PPV1-PPV7=PCR result of positive control with each primer pair from PPV1 to PPV7, Sample PPV1-PPV7=PCR result of sample with each primer pair from PPV1 to PPV7. PPV=Porcine parvovirus, PCR=Polymerase chain reaction.

Thirty-two samples (29 serums and 3 stillbirths) were identified as PPV-positive using the PCR assay. The specific PCR product size was 330 bp (Figure-2). PPV-positive samples from Thaibinh, Thanhhoa, Binhdinh, Daklak, Dongnai, and Binhduong were obtained as 5, 3, 4, 6, 7, and 7 samples, respectively (Table-1). All 32 samples were detected as PPV1 (Figure-3).

**Table-1:** PPV positivity rate according to the sample collection location.

Location	Total samples	PPV DNA positive	Percentage
Thaibinh	60	5	8.3
Thanhhoa	63	3	4.8
Binhdinh	60	4	6.7
Daklak	69	6	8.7
Dongnai	70	7	10
Binhduong	70	7	10
Total	392	32	8.2

PPV=Porcine parvovirus

Thanhhoa had the lowest PPV-positive rate of 4.8%. The highest PPV-positive rates are in Dongnai and Binhduong (10%) (Table-1). The PPV-positive rates in serum and stillbirth samples are similar at 9.1% and 9.4%, respectively. Sows have a higher PPV infection rate than gilts (9.4% and 4.6%, respectively) (Table-2).

**Table-2:** PPV positivity rate according to the sample type or maternal pig.

Location	Maternal serum		Stillbirth		Gilt		Sow	
			
	Total (%)	PPV DNA positive (%)	Total (%)	PPV DNA Positive (%)	Total (%)	PPV DNA positive (%)	Total (%)	PPV DNA positive (%)
Thaibinh	60	5/60	0	0	20	1/20	40	4/40
	100	8.3	0	0	100	5	100	10
Thanhhoa	60	3/60	3	0/3	18	0/18	45	3/45
	100	5	100	0	100	0	100	6.7
Binhdinh	60	4/60	0	0	15	0/15	45	4/45
	100	6.7	0	0	100	0	100	8.9
Daklak	60	5/60	9	1/9	19	1/19	50	5/50
	100	8.3	100	11.1	100	5.3	100	10
Dongnai	60	5/60	10	2/10	17	1/17	53	6/53
	100	8.3	100	20	100	5.9	100	11.3
Binhduong	60	7/60	10	0/10	20	2/20	50	5/50
	100	11.7	100	0	100	10	100	10
Total	360 (100)	29/360 (9.1)	32 (100)	3/32 (9.4)	109 (100)	5/109 (4.6)	283 (100)	27/283 (9.5)

PPV=Porcine parvovirus

### Parvovirus isolation

32 PPV-positive samples tested negative for PRRS, CSF, and PCV. Virus isolation was attempted with PK-15 cells. CPE was observed daily for 5 days [6, 22]. 3–5 days after incubation, CPE was observed in all 32 samples, with cells rounding, clustering, condensing, then disintegrating and losing shape. The cells broke free from the bottle wall (Figure-5). Real-time PCR was used alongside CPE observations to measure the viral copy number in cultures.

**Figure-4:**
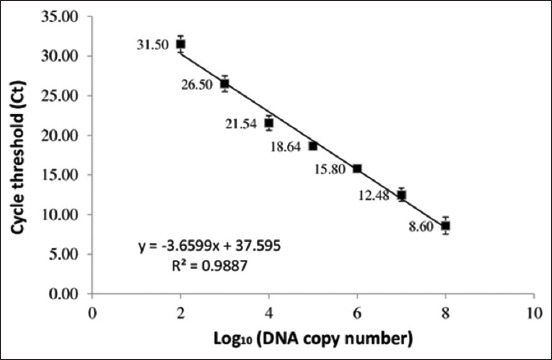
Standard curve for the quantification of viral copy number by real-time polymerase chain reaction.

**Figure-5:**
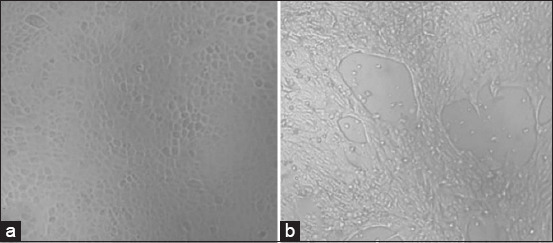
Cytopathic effects appeared on PK15 cells at 5 days after infection with PPV. (a) PK15 cells control, no virus, (b) PK15 cells with PPV infection. PPV=Porcine parvovirus.

The real-time PCR test revealed an average Ct value of 25.46 for the first culture, 20.11 for the second, and 18.76 for the third. In Figure-4, the virus copy numbers in the first, second, and third cultures were 10^3.3^, 10^4.8^, and 10^5.1^ copies/μL, respectively, as indicated by the standard curve. With each culture transfer, the virus multiplies. The virus was successfully cultured, as evidenced by this result.

All 32 PPV strains were successfully isolated. The TCID_50_ titers of isolates ranged from 4.5log_10_ TCID_50_/1 mL to 6.3log_10_ TCID_50_/1 mL. VC5 and TX7 had the highest TCID_50_ titers.

### VP2 phylogenetic tree

**Figure-6:**
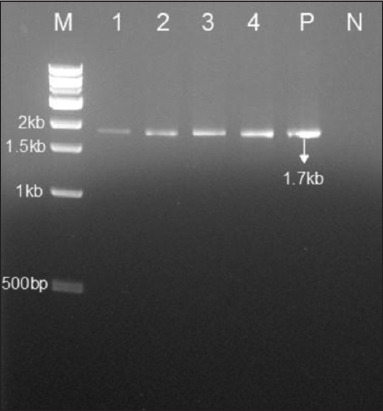
Polymerase chain reaction for VP2 gene detection. M=1kb deoxyribonucleic acid ladder, 1-4=Samples, P=Positive control, N=Negative control.

The VP2 gene, which encodes the capsid protein was amplified by PCR from the PPV genomic DNA. The VP2 fragment was 1760 bp in length (Figure-6).

**Figure-7:**
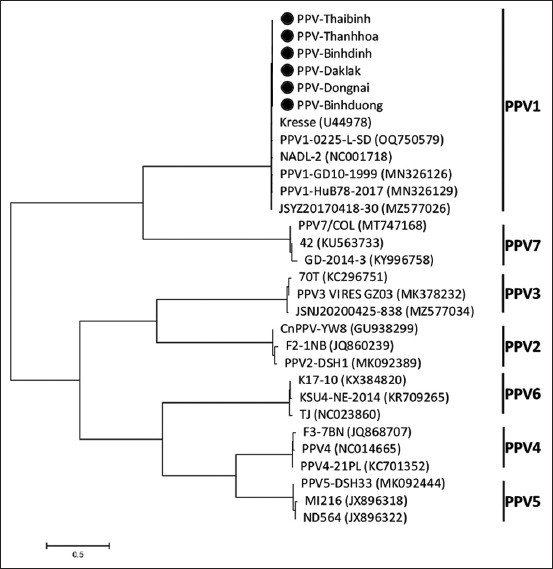
Phylogenetic tree constructed based on deoxyribonucleic acid sequences of the VP2 gene.

Based on phylogenetic analysis, all strains had identical traits and were closely related to PPV1 sequences. Six sequences from six provinces were selected to be uploaded to the GenBank National Center for Biotechnology Information (Accession numbers: OR263486–OR263491). The phylogenetic tree shows the closest relationship of these strains to the Kresse strain (from Canada in 1996) and the PPV1-0225-L-SD (from China in 2022) (Figure-7).

### Virulence in gilts

VC5 and TX7, which had the highest TCID_50_ titers, were selected for the group 1 and 2. The results of HI antibody titers on days 0, 7, 14, 21, and 49 after infection are shown in Table-1. In groups 1 and 2, all gilts developed HI titers against PPV. The HI antibody titers were 4log_2_–6log_2_ on Day 7, 7log_2_–9log_2_ on Day 14, 10log_2_–12log_2_ on Day 21, and 9log_2_–10log_2_ on Day 49 (Table-3). All gilts were negative for PPV antibodies in group 3 (control group).

**Table-3:** HI antibody titers of the pregnant sows after virus injection.

Group	Virus strain	Gilt No.	HI titer of maternal serum on day (log_2_)				

			Day 0	Day 7	Day 14	Day 21	Day 49
1	TX7	1	0	5	8	11	10
		2	0	6	9	12	10
		3	0	5	8	11	10
2	VC5	4	0	5	8	11	10
		5	0	5	8	11	10
		6	0	4	7	10	9
3	No virus	7	0	0	0	0	0
		8	0	0	0	0	0
		9	0	0	0	0	0

HI=Hemagglutination inhibition

**Figure-8:**
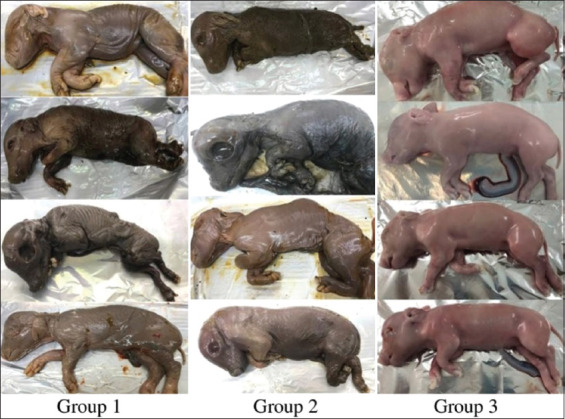
The fetuses were euthanized at day 90 after virus injection. Group 1: Pigs were inoculated with PPV-TX7 strain. Group 2: Pigs were inoculated with PPV-VC5 strain. Group 3: Pigs were inoculated with PBS (control group). PPV=Porcine parvovirus, PBS=Phosphate buffered saline.

The virulence of VC5 and TX7 for pregnant gilts following experimental infection is presented in Table-4. The virus infection group had a higher fetal mortality rate than the control group. A total of 19 fetuses in the control group were alive and unaffected. 100% of fetuses in groups 1 and 2 were dead. 71%–80% of the fetuses in groups 1 and 2 were mummified. Fetuses turned from yellowish-brown to black. In comparison to the control group, the fetus was both under-weighted and dry (Figure-8).

**Table-4:** Vitality and presence of PPV in fetuses per sow.

Group	Virus strain	Gilt No.	HI titer of maternal serum on day (log_2_)				PPV DNA Pos (PCR)

			Total	Alive	Dead		

					Non-mummified	Mummified	
1	TX7	1	5	0	1	4	5
		2	6	0	2	4	5
		3	6	0	2	4	5
Total			17	0	5	12	15
Percentage			100	0	29	71	88
2	VC5	4	7	0	1	6	7
		5	5	0	1	4	5
		6	8	0	2	6	6
Total			20	0	4	16	18
Percentage			100	0	20	80	90
3	No virus	7	8	8	0	0	0
		8	6	6	0	0	0
		9	5	5	0	0	0
Total			19	19	0	0	0
Percentage			100	100	0	0	0

PPV=Porcine parvovirus, HI=Hemagglutination inhibition, PCR=Polymerase chain reaction

Approximately 88%–90% of placental PPV DNAs in virus-infected fetuses were detected (Table-4). The virus was found in the fetus indicating prenatal transmission from the mother.

## Discussion

PPV1, first identified in 1967 [1], is now widespread among swine herds in every nation [3, 4, 6]. Recently, many new PPV1 strains with genetic variations were discovered [23, 24]. For example, in 2024, two new PPV1 (HLJ202108-Y and SDLC202109) from northern China were found to have three amino acid substitutions (K195R, K562R, and S578P) in NSP 1 [24]. This study provides the first description of PPV strains in Vietnam, while previous investigations focused on detecting the virus through PCR or antibody testing [25, 26]. PPV1 is the most prevalent genotype among current pig isolates in various countries. 88.2% (60/68) of livestock farm serum samples in six provinces (Hanoi, Hungyen, Haiduong, Bacninh, Hoabinh, and Vinhphuc) in 2020 contained PPV1 antibodies [26]. Ten samples were confirmed as PPV1 through genetic analysis using the partial NS1- protein-coding gene [26]. Using PCR, 8.2% of the samples tested positive for PPV (Table-1). A study by Nguyen *et al*. [25] found PPV infection rates ranging from 24.4% to 61.6%, while Thuy *et al*. [13] found ranging from 3% to 65%. However, these infection rates were only based on samples from slaughterhouses [13] or samples that tested positive for another virus [25]. Since the slaughterhouses typically contain meat pigs and sows that are unable to reproduce and the challenging slaughter conditions impair sterility verification [27], the research findings might not reflect the precise prevalence of PPV in Vietnam. We found positive PPV rates on pig samples randomly chosen for producing piglets. By collecting samples from farms, we can more accurately determine the PPV infection rate affecting the reproductive rates of pigs. The PPV infection rate of this study is comparable to previous studies that used similar methods to identify PPV-positive samples in Argentina (12.97%) [28] and South India (14.3%) [29].

Using the neighbor-joining method, a phylogenetic tree of VP2 sequences from porcine parvoviruses found in Vietnam was constructed to study their genetic relationships. VP2 gene sequencing results did not distinguish among PPV strains circulating in Vietnam. The phylogenetic tree shows a close relationship between these strains and the Kresse strain (1996, Canada) and PPV1-0225-L-SD (2022, China) (Figure-7). A ”Kresse-like” K22 PPV strain-based vaccine offered more effective protection than the NADL-2 and NADL-like strain-based commercial vaccines in a recent study against a PPV-27a cluster strain challenge [11]. Selecting strains for vaccine development should consider their close relationship with the Kresse strain identified in this study.

The titers of HI antibodies in TX7 and VC5 reached their highest levels (10log_2_–12log_2_) on day 21 following the challenge and continued to last for up to 49 days as shown in Table-3. The findings from this study agree with those of another research indicating the ability to detect PPV-specific antibodies by day 6 post-infection, with peak concentrations occurring on day 21 post-infection [30]. HI titers against PPV ranged from 11log_2_ to 13log_2_ after 8–50 days of infection [31]. 100% of the fetuses died and 71%–80% of them were mummified after the pregnant gilts were infected with VC5 and TX7 viruses. (Table-4). Only 5%–18% of fetuses were mummified in the investigation on PPV virulence in pigs, which showed lower pathogenicity of the PPV-143a, PPV-IDT, and PPV-NADL-2 strains [21]. According to the study findings, TX7 and VC5 are proposed as possible vaccine candidates.

## Conclusion

The current study marks Vietnam’s first successful isolation of PPV strains. This study details the characterization, virulence, prevalence, and classifications of Vietnamese strains of PPV. 32 isolates from Vietnam’s PPV infections were all PPV1 type. These strains share the closest relationship with the Kresse strain from Canada (1996) and the Chinese PPV1-0225-L-SD strain (2022). The highly virulent PPV strains, VC5 and TX7, were identified through experimental injections in gilts. This study revealed VC5 and TX7, two Vietnamese PPV strains, as promising options for the creation of affordable and efficient vaccines. The data from this study are critical for creating a PPV vaccine in Vietnam, as domestic vaccines are yet to be available. Developing a vaccine against PPV in pigs using Vietnamese isolates will cost-effectively and effectively replace imported vaccines.

## Authors’ Contributions

TTHT: Conceptualization, methodology, and drafted and revised the manuscript. VTD and VKD: Data collection and curation and writing-original draft. HV: Methodology, supervision, and project administration. All authors have read, reviewed, and approved the final manuscript.

## References

[1] Cartwright S.F, Huck R.A (1967). Viruses isolated in association with herd infertility, abortions and stillbirths in pigs. Vet. Rec.

[2] Streck A.F, Truyen U (2020). Porcine parvovirus. Curr. Issues Mol. Biol.

[3] Faustini G, Tucciarone C.M, Franzo G, Donneschi A, Boniotti M.B, Alborali G.L, Drigo M (2024). Molecular survey on *Porcine parvoviruses* (PPV1-7) and their association with major pathogens in reproductive failure outbreaks in Northern Italy. Viruses.

[4] Wang D, Mai J, Yang Y, Wang N (2020). *Porcine parvovirus* 7:Evolutionary dynamics and identification of epitopes toward vaccine design. Vaccines (Basel).

[5] Vargas-Bermudez D.S, Mogollon J.D, Franco-Rodriguez C, Jaime J (2023). The novel *Porcine parvoviruses*:Current state of knowledge and their possible implications in clinical syndromes in pigs. Viruses.

[6] Streck A.F, Homeier T, Foerster T, Fischer S, Truyen U (2013). Analysis of *Porcine parvoviruses* in tonsils and hearts from healthy pigs reveals high prevalence and genetic diversity in Germany. Arch. Virol.

[7] Xu Y.G, Li Y.J (2007). Induction of immune responses in mice after intragastric administration of *Lactobacillus casei* producing *Porcine parvovirus* VP2 protein. Appl. Environ. Microbiol.

[8] Kong M, Peng Y, Cui Y, Chang T, Wang X, Liu Z, Liu Y, Zhu Y, Luo Y, Tang Q, Feng L, Cui S (2014). Development and evaluation of the rVP-ELISA for detection of antibodies against *Porcine parvovirus*. J. Virol. Methods.

[9] Soares M.R, Cortez A, Heinemann M.B, Sakamoto S.M, Martins V.G, Bacci M, De Campos Fernandes F.M, Richtzenhain L.J (2003). Genetic variability of *Porcine parvovirus* isolates revealed by analysis of partial sequences of the structural coding gene VP2. J. Gen. Virol.

[10] Oh W.T, Kim R.Y, Nguyen V.G, Chung H.C, Park B.K (2017). Perspectives on the evolution of *Porcine parvovirus*. Viruses.

[11] Kiss I, Kovács E, Zádori Z, Mészáros I, Cságola A, Bajnóczi P, Mortensen P, Palya V (2020). Vaccine protection against experimental challenge infection with a PPV-27a genotype virus in pregnant gilts. Vet. Med. (Auckl).

[12] Chu X.L, Zhang B.W, Zhang Q.G, Zhu B.R, Lin K, Zhang D.Y (2018). Temperature responses of mutation rate and mutational spectrum in an *Escherichia coli* strain and the correlation with metabolic rate. BMC Evol. Biol.

[13] Thuy N.T.D, Trung N.T, Dung T.Q, Khoa D.V.A, Thuy D.T.N, Opriessnig T (2021). First investigation of the prevalence of parvoviruses in slaughterhouse pigs and genomic characterization of ungulate *copiparvovirus* 2 in Vietnam. Arch. Virol.

[14] Lyoo K.S, Park Y.H, Park B.K (2001). Prevalence of porcine reproductive and respiratory syndrome virus, porcine circovirus type 2 and *Porcine parvovirus* from aborted fetuses and pigs with respiratory problems in Korea. J. Vet. Sci.

[15] Kim S.C, Jeong C.G, Nazki S, Lee S.I, Baek Y.C, Jung Y.J, Kim W.I (2021). Evaluation of a multiplex PCR method for the detection of *Porcine parvovirus* types 1 through 7 using various field samples. PLoS One.

[16] Miao L.F, Zhang C.F, Chen C.M, Cui S.J (2009). Real-time PCR to detect and analyze virulent PPV loads in artificially challenged sows and their fetuses. Vet. Microbiol.

[17] Xu Y.G, Cui L.C, Wang H.W, Huo G.C, Li S.L (2013). Characterization of the capsid protein VP2 gene of a virulent strain NE/09 *of Porcine parvovirus* isolated in China. Res. Vet. Sci.

[18] Saitou N, Nei M (1987). The neighbor-joining method:A new method for reconstructing phylogenetic trees. Mol. Biol. Evol.

[19] Felsenstein J (1985). Confidence limits on phylogenies:An approach using the bootstrap. Evolution.

[20] Ramakrishnan M.A (2016). Determination of 50% endpoint titer using a simple formula. World J. Virol.

[21] Zeeuw E.J.L, Leinecker N, Herwig V, Selbitz H.J, Truyen U (2007). Study of the virulence and cross-neutralization capability of recent *Porcine parvovirus* field isolates and vaccine viruses in experimentally infected pregnant gilts. J. Gen. Virol.

[22] Mengeling W.L (1972). *Porcine parvovirus*:Properties and prevalence of a strain isolated in the United States. Am. J. Vet. Res.

[23] Vereecke N, Kvisgaard L.K, Baele G, Boone C, Kunze M, Larsen L.E, Theuns S, Nauwynck H (2022). Molecular epidemiology of *Porcine parvovirus* type 1 (PPV1) and the reactivity of vaccine-induced antisera against historical and current PPV1 strains. Virus. Evol.

[24] Deng H, Cong G, Wang H, Hu Z, Shi D, Shi H, Xia C, Fu F, Feng L (2024). Isolation, characterization, and phylogenetic analysis of two new *Porcine parvovirus* 1 isolates from Northern China. Virus Res.

[25] Nguyen V.G, Dang H.A, Nguyen T.T, Huynh T.M.L, Nguyen B.H, Pham L, Le H.T.P (2022). Polymerase chain reaction-based detection of coinfecting DNA viruses in Vietnamese pigs in 2017 and 2021. Vet. World.

[26] Nguyen V.G, Mai T.N, Le V.T, Vu T.N, Vo V.H, Ta,T.K.C. Vu, D.H, Huynh T.M.L (2020). The presence of *Porcine parvovirus* 1 (PPV1) in pork in Hanoi and surrounding areas. Vietnam J. Agric. Sci.

[27] Dang-Nguyen T.Q, Tich N.K, Nguyen B.X, Ozawa M, Kikuchi K, Manabe N, Ratky J, Kanai Y, Nagai T (2010). Introduction of various Vietnamese indigenous pig breeds and their conservation by using assisted reproductive techniques. J. Reprod. Dev.

[28] Serena M.S, Cappuccio J.A, Metz G.E, Aspitia C.G, Dibárbora M, Calderón M.G, Echeverría M.G (2019). Detection and molecular characterization of *Porcine parvovirus* in fetal tissues from sows without reproductive failure in Argentina. Heliyon.

[29] Parthiban S, Sowndhraya R.K.V, Raja P, Parthiban M, Ramesh A, Dhinakar Raj G, Senthilkumar K, Balasubramanyam D, Hemalatha S, Bharathi R, Ravishankar C, Thahira Parveen S (2022). Molecular detection of *Porcine parvovirus* 1-associated reproductive failure in southern India. Trop. Anim. Health Prod.

[30] Mészáros I, Olasz F, Cságola A, Tijssen P, Zádori Z (2017). Biology of *Porcine parvovirus* (ungulate parvovirus 1). Viruses.

[31] Van den Born E, Van den Elzen P.P.M, Van Kilsdonk E, Hoeijmakers M.J.H, Segers R.P.A.M (2020). An octavalent vaccine provides pregnant gilts protection against a highly virulent *Porcine parvovirus* strain. BMC Vet. Res.

